# Dose-Dependent Effects of Replacing Soybean Meal with Cottonseed Protein: Key to Optimizing Gut Health in Weaned Piglets

**DOI:** 10.3390/ani16060946

**Published:** 2026-03-18

**Authors:** Hewei Jin, Aiwen Zhang, Linna Xu, Defu Tang, Shizhen Qin

**Affiliations:** 1 College of Animal Science and Technology, Gansu Agricultural University, Lanzhou 730070, China; 18394432985@163.com (H.J.); tangdf@gsau.edu.cn (D.T.); 2 Gansu Provincial Livestock Technology Promotion Station, Lanzhou 730000, China; 18394036036@163.com (A.Z.); 15909642341@163.com (L.X.)

**Keywords:** cottonseed protein, weaned piglets, growth performance, gut health

## Abstract

This study aims to systematically assess the comprehensive, dose-dependent effects of substituting soybean meal with cottonseed protein at various ratios on weaned piglets. In total, 28-day-old weaned piglets (Duroc × Landrace × Large White crossbred; *n* = 45) were selected and then randomly categorized into three groups: 100% soybean meal (CON), 50% soybean meal +50% cottonseed protein (CSP50), and 100% CSP (CSP100) groups. After a 7-day adaptation period, the experiment continued for an additional 28 days. The results showed no significant differences among groups in growth performance, organ indices, most carcass traits, or meat quality indicators. The CSP50 group showed significantly reduced levels of diamine oxidase (DAO) and D-lactate and increased complexity of the colonic microbial network, with improved abundance of beneficial bacterial genera such as *g_Blautia* and *g_Eubacterium*. The CSP100 group showed elevated intestinal permeability, a decreased villus height, a villus-to-crypt ratio, specific digestive enzymes, a reduced Firmicutes/Bacteroidetes (F/B) ratio and abundant inflammation-associated bacteria, including *g_Streptococcus.* Furthermore, correlation analysis suggested that specific gut microorganisms and metabolic pathways may be potentially related to average daily gain (ADG), average daily feed intake (ADFI), the feed conversion ratio (F/G), DAO, and D-lactic acid. These findings suggest that dietary inclusion of 50% cottonseed protein (CSP50) is associated with sustained growth performance and enhanced gut health in weaned piglets, concurrent with shifts in the composition and predicted function of the gut microbiota.

## Simple Summary

This study addressed the issue of excessive reliance on expensive soybean meal in livestock farming by exploring the feasibility of using abundant, low-cost cottonseed protein as an alternative protein source. Given that cottonseed protein contains substances like gossypol that may damage the intestines of young animals, the research aimed to clarify the comprehensive effects of different substitution ratios on weaned piglets. The results indicate that replacing half of the soybean meal with cottonseed protein allows piglets to grow normally while enhancing intestinal protective capacity (significantly reduced levels of DAO and D-lactate), increasing beneficial bacteria (*g_Blautia* and *g_Eubacterium*), and stabilizing the gut microbiota network. However, complete replacement with cottonseed protein may impair the intestinal structure and increase harmful bacteria. Therefore, while cottonseed protein is a viable soybean meal substitute, moderate replacement ratios are recommended to balance animal health with feed cost reduction, thereby promoting sustainable development in the livestock industry.

## 1. Introduction

Weaning is a pivotal stage during which piglets encounter various stressors, including the transition from easily digestible colostrum to plant-based solid nutrition, alterations in their surroundings, and reorganization of social groups. These variables exacerbate their physiological challenges [1]. During this phase, the nutritional quality of dietary protein is essential. The source not only affects the morphological structure of the piglet’s small intestine but also directly governs feed intake. Unsuitable protein sources can readily induce diarrhea in piglets and decrease their appetite [2].

Globally, soybean meal is employed as the primary protein source for swine [3]. Although the livestock industry is highly dependent on large-scale soybean meal imports (Brazil, the United States, Argentina, etc.), environmental factors and increasing costs have rendered its use unsustainable [4,5]. To decrease the livestock industry’s dependence on conventional protein sources like soybean meal, while addressing the shortage of animal feed and perhaps reducing expenses, the use of alternative feeds is a sustainable solution for protein sources in swine diets [6]. The currently available alternative protein sources include plant- and animal-based proteins, such as rapeseed meal [7], peanut meal [8], and broad beans [9]; as well as new potential protein sources, including insect protein [10], fermented soybean meal, and soybean concentrate protein [11]. However, cottonseed protein, as a plentiful and relatively low-cost potential protein source, is often underestimated compared to other alternatives.

Cottonseed protein (CSP) is a plentiful and relatively inexpensive potential protein source and is undervalued relative to other alternatives. It is a high-value plant protein extracted by pressing cottonseed protein. Its quality exceeds that of cottonseed cake meal and is analogous to soybean meal. CSP comprises approximately 50% crude protein, surpassing soybean meal, and its arginine content exceeds that of other cake meal basic materials; however, its lysine level is significantly lower than that of soybean meal [12]. CSP, a plant-derived protein source with elevated protein levels, can effectively mitigate the existing deficit of feed protein resources when utilized in the feed industry. Due to the presence of anti-nutritional components, such as free gossypol in CSP, its use in animal feed has been predominantly focused on aquaculture [13]. Only a few studies have reported the application of CSP in weaned piglets [14]. However, if the concentrations of anti-nutritional components in CSP decreased to acceptable levels, it can be employed as a protein source for the swine industry. Therefore, the selection of three replacement levels 0%, 50%, and 100% to substitute soybean meal with CSP is based on the following: the 0% control group (100% soybean meal) serves as the baseline, representing conventional commercial feed; The 50% replacement rate represents an intermediate level, which may balance the risk of CSP antinutritional factors; the 100% replacement aims to explore the upper limit of CSP addition and assess whether complete soybean meal replacement induces detectable intestinal barrier dysfunction or microbial dysbiosis.

This study aims to investigate the effects of replacing soybean meal with CSP at different ratios on the diets of weaned piglets. Therefore, CSPs’ impact on growth performance, carcass traits, organ indices, meat quality, intestinal barrier function, intestinal morphology, and the intestinal microbiota’s structure and function in weaned piglets was also assessed. The findings will provide evidence for the livestock industry to utilize CSP as a novel protein feed ingredient.

## 2. Materials and Methods

### 2.1. Experimental Design and Feeding Management

This experiment was conducted at the Tianshui Dongcha Experimental Base. Forty-five 28-day-old “Duroc × Landrace × Large White” crossbred weaned piglets (castrated males) in good health with an initial weight of 9.62 ± 0.45 kg were selected. After a 7-day pre-trial period, the pigs were randomly divided into 3 groups with 5 replicates (pen) and 3 piglets. The control group (CON) was fed a diet containing 100% soybean meal as the protein source; the experimental group I (CSP50) was fed a diet containing 50% cottonseed protein and 50% soybean meal as protein sources; the experimental group II (CSP100) was fed a diet containing 100% cottonseed protein as the protein source. The free gossypol content in cottonseed protein is relatively low, controlled at 200–600 mg/kg. The basal diet was formulated according to NRC (2012) standards [15], with its composition shown in Table 1.

Piglets are housed with duckbill drinkers and adjustable stainless-steel feeders to ensure free access to feed and water. Nursery pens measure 2.2 m × 1.8 m with hard plastic slatted flooring. Indoor humidity and temperature are maintained at 60–70% and 24–26 °C, respectively. Throughout the experiment, daily health observations are conducted, and routine vaccinations and deworming are administered according to farm management protocols.

### 2.2. Sample Collection

On the 28th day of the experiment, blood samples were obtained from the anterior vena cava of the test pigs via vacuum-activated clotting tubes. After a standing period of 1–2 h, the tubes were centrifuged at 1000× *g* for 10 min to collect serum, which was aliquoted and preserved at −20 °C for further examination. After blood collection, piglets were euthanized by intramuscular administration of pentobarbital sodium (50 mg/kg body weight). Then, the abdominal cavity was excised, and the viscera, intestines, and longissimus dorsi muscle were separated. Subsequently, two 2 cm segments from the central regions of the duodenum, jejunum, and ileum were collected. One segment was rinsed with phosphate-buffered saline (PBS), placed in a cryovial, quickly frozen in liquid nitrogen, and stored at −80 °C for subsequent analysis, while the other piece was fixed in a 4% paraformaldehyde. Furthermore, content was also harvested from the mid-colonic segment, transferred to a cryovial, and preserved in liquid nitrogen.

### 2.3. Growth Performance

Daily mortality was recorded in each group. Each pig was weighed on an empty stomach (after 12 h fasting) at 8:00 a.m. on day 1 (initial body weight: IBW), 14, and 28 (final body weight: FBW) to calculate ADG. Furthermore, daily feed intake of each piglet was also recorded to calculate average ADFI. Then, the F/G was measured based on the ADFI and ADG of the experimental pigs. The calculation formulas are as follows:

ADG = (FBW − IBW)/(Number of Days × Number of Animals);

ADFI = Total Feed Intake/(Number of Days × Number of Animals);

F/G = ADFI/ADG.

### 2.4. Measurement of Carcass Characteristics

Euthanize and dissect the animals, then conduct carcass trait measurements in accordance with the Chinese agricultural industry standard “Technical Specifications for Carcass Trait Measurement of Lean Pigs” (NY/T 825-2004 [16]), namely carcass weight, carcass yield, carcass straight length, carcass oblique length, average backfat thickness, and eye muscle area.

### 2.5. Organ Index

To calculate the organ index, the heart, liver, spleen, lungs, and kidneys were dissected, their blood was blotted away using filter paper, fatty tissues were removed, and then the organ weight was assessed. Organ index was calculated as follows:

Organ Index = Organ Weight (g)/Pre-slaughter Live Weight (kg)

### 2.6. Meat Quality Assessment

For meat quality assessment, the Chinese agricultural industry standard “Technical Specifications for Pork Quality Determination” (NY/T 821-2019 [17]) suggests that the longest back muscle should be extracted within 20 min post-slaughter.

### 2.7. Intestinal Permeability, Morphology, and Mucosal Digestive Enzyme Activity

Serum diamine oxidase activity was measured by following the method provided in an enzyme-linked immunosorbent assay (ELISA) kit (Jiangsu Kete Biotechnology Co., Ltd., Nanjing, Jiangsu, China). Serum D-lactate levels were determined using a D-lactate assay kit (Jiangsu Kete Biotechnology Co., Ltd., Nanjing, Jiangsu, China).

Duodenal, jejunal, and ileal tissues were collected; fixed in 4% paraformaldehyde; dehydrated; cleared; embedded in paraffin; and sectioned. The sections were then stained with hematoxylin and eosin (HE), and imaged at 20× magnification via the BA210 microscope system (Macau Industrial Group Co., Ltd., Macau, China). The Image-Pro Plus 6.0 analysis software was employed to assess the villus height (VH) and crypt depth (CD) in micrometers. Histological measurements were performed blinded to treatment. Three sections per pig were analyzed, 5 complete VH and 5 CDs were measured per section, and the villus-to-crypt ratio (V/C) = VH/CD was calculated.

Piglet duodenal, jejunal, and ileal mucosal samples were thoroughly assessed at a 1:9 weight-to-volume ratio (e.g., 1 g tissue sample to 9 mL PBS) and centrifuged at 3000× *g* for 10 min at 4 °C to collect the supernatant. Activities of digestive enzymes in the duodenal, jejunal, and ileal mucosa were assessed using kits for invertase (KT8195-B), maltase (KT84018-B), lactase (KT84016-B), trypsin (KT8195-B), lipase (ADS-F-ZF005), and α-amylase (ADS-F-TDX067). All kits were acquired from Jiangsu Kete Biotechnology Co., Ltd. Sucrase, maltase, lactase, and trypsin enzyme activities are expressed in U/mL or U/L, with the denominator parameter being protein concentration. Alpha-amylase activity is defined as the amount of enzyme that hydrolyzes 10 mg of starch per gram of tissue at 37 °C over 30 min, defined as one enzyme activity unit. Lipase activity is defined as one enzyme activity unit per gram of tissue per minute releasing 1 nmol of p-nitrophenol.

### 2.8. DNA Extraction and Sequencing

Metagenomic sequencing analysis of gut microbiota was performed using colonic chyme from weaned pigs. Briefly, DNA was extracted with the MagBeads FastDNA Kit for Soil (116564384) (MP Biomedicals, Irvine, CA, USA) and then quantified using the Qubit™ 4 Fluorometer (Qubit 4.0; Thermo Fisher Scientific, Singapore). Then, DNA purity was assessed using 1% agarose gel electrophoresis. DNA samples that met quality standards were fragmented with a Bioruptor machine (Shuangjia Biological, Ningbo, Zhejiang, China), repaired utilizing the combined action of 3’–5’ exonucleases and polymerases in EndRepairMix, ligated by the action of ligase, using magnetic beads to remove free and self-ligated adapter sequences while selectively purifying DNA fragments of appropriate size, and amplified DNA library. Libraries were measured using Qubit4 and assessed for quality using PCR-amplified fragment analysis with Agilent 2100 (Santa Clara, CA, USA) to confirm DNA library fragment size and distribution. Metagenomic sequencing was conducted with Illumina NovaSeq/HiSeq high-throughput sequencing systems (San Diego, CA, USA).

### 2.9. Sequence Analysis

To analyze the sequence, the R software package (v 4.3.3) was employed. The relative abundance of microbial taxonomic units was visualized using Origin 2024 software. Alpha diversity indices at the operational taxonomic unit (OTU) level were assessed from OTU tables within the Quantitative Insights into Microbial Ecology (QIIME) software. Furthermore, for beta diversity analysis, Bray–Curtis distance metrics were utilized, which were visualized via principal coordinate analysis (PCoA) to elucidate microbial community composition across samples. Moreover, the Personalbio tool (https://www.genescloud.cn/ (accessed on 10 March 2026)) was employed for the analysis of species-level differences. Gut microbiota functions were annotated via the Kyoto Encyclopedia of Genes and Genomes (KEGG) database, with heatmaps generated with Omicshare tools (version 4.0, https://www.omicshare.com/ (accessed on 10 March 2026)). For correlation analyses, the Personalbio tool (https://www.genescloud.cn/ (accessed on 10 March 2026)) was employed, whereas for co-occurrence network construction and topological parameter calculation, the R package digraph (version 1.5.1) was used. Networks were visualized using Gephi software (version 0.9.2).

### 2.10. Statistical Analysis

Data recorded during the experiment were preliminarily organized using Excel software. Following normality and homogeneity of variance tests conducted with SPSS 27.0 software, one-way ANOVA and linear and quadratic trend analyses were performed. Multiple comparisons were analyzed using the LSD method. * *p* < 0.05, ** *p* < 0.01, *** *p* < 0.001.

## 3. Results

### 3.1. Effects of Replacing Soybean Meal with CSP on Growth Performance, Carcass Characteristics, Organ Indices, and Meat Quality in Weaned Piglets

There was no significant difference in IBW (*p* > 0.05), and after 28 days of feeding, the CSP50 and CSP100 groups showed no significant effects on FBW, ADG, ADFI, and F/G compared to the CON (*p* > 0.05; Table 2). Furthermore, no significant differences (*p* > 0.05) were found among groups in carcass yield, carcass straight length, carcass oblique length, average backfat thickness, and eye muscle area (Table 3). However, the CON indicated significantly higher carcass weight relative to the CSP50 and CSP100 groups (*p* = 0.014). Moreover, there were no substantial differences in organ indices (heart, liver, spleen, lung, and kidney) among the groups (*p* > 0.05; Table 4). Further, the differences in pH, cooking water loss, shear force, and drip loss among groups were not significant (*p* > 0.05; Table 5). Except for L* 45 min (*p* = 0.002) and b* 24 h (*p* = 0.005), which were significantly higher in the CSP50 and CSP100 groups compared to the CON.

### 3.2. Effects of Replacing Soybean Meal with CSP on Intestinal Permeability, Intestinal Morphology, Mucosal Enzyme Activity in Weaned Piglets

**Figure 1:**
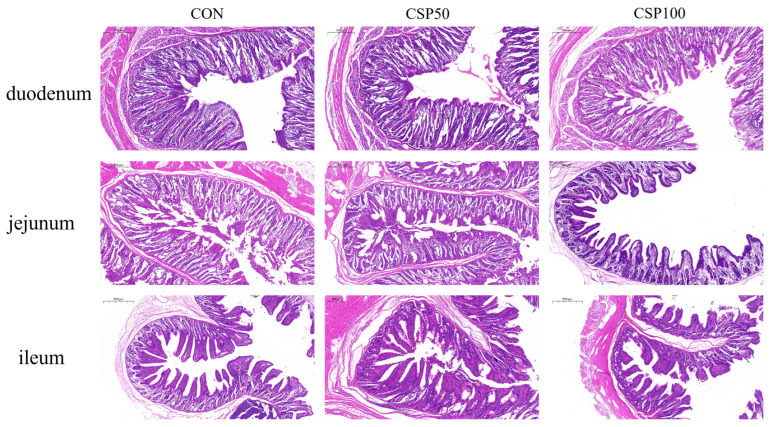
Tissue morphology of the duodenum, jejunum, and ileum.

Compared to the CON, the serum DAO and D-lactate contents of the CSP50 group were significantly reduced *(p* < 0.001; Table 6), but they were significantly elevated in the CSP100 group. Furthermore, relative to the CON and CSP50 groups, the CSP100 group showed a substantial reduction in VH and V/C ratios in the duodenum and ileum (*p* < 0.05, Figure 1, Table 7), while a significant increase in CD was observed in the jejunum (*p* = 0.033). Moreover, the CON and CSP50 groups indicated substantially higher lipase activity in duodenal mucosa (*p* < 0.001) and lactase activity in jejunum (*p* < 0.001) than the CSP100 group (Table 8). The CSP50 group’s invertase activity in the duodenum and jejunum (*p* = 0.018 and *p* < 0.001, respectively) and the maltase activity in the ileum were substantially higher than those in the CON and CSP100 groups (*p* = 0.017).

### 3.3. Composition and Diversity of Microbial Species in the Colonic Content

**Figure 2:**
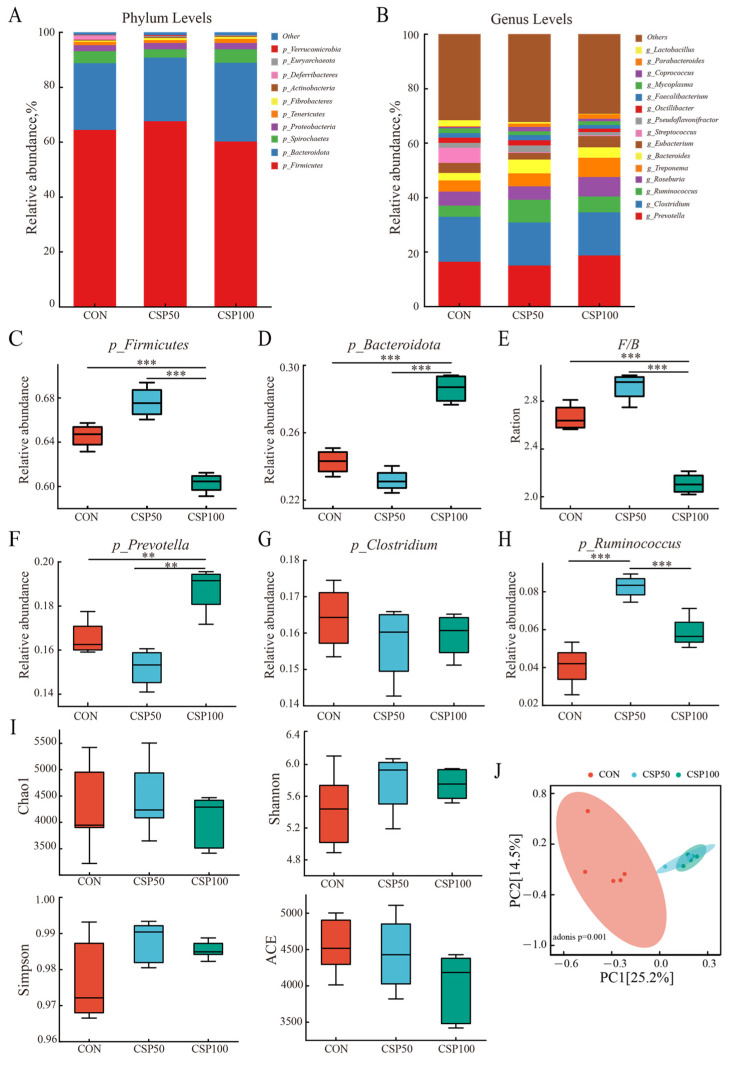
Composition and diversity of microbial species in the colonic content. (**A**) The distribution map of colonic content microbiota at the phylum level and (**B**) the genus level. (**C**–**E**), The relative abundance of *Firmicutes* (**C**), *Bacteroidota* (**D**), F/B (**E**), *Prevotella* (**F**), *Clostridium* (**G**) and *Ruminococcus* (**H**) in colonic content microbiota in piglets. (**I**) The α diversity of the colonic content microbiota was assessed using the Chao1, Shannon, Simpson, and ACE indices. (**J**) The β diversity of the colonic content microbiota. ** *p* < 0.01, *** *p* < 0.001.

Statistical analysis at the phylum level indicated that Firmicutes and Bacteroidetes were the predominant microbial phyla in the colon, comprising over 80% of the overall abundance (Figure 2A). Compared to the CON and CSP50 groups, the CSP100 group had a considerably reduced relative abundance of Firmicutes and a significantly elevated relative abundance of Bacteroidetes (Figure 2C,D, *p* = 0.005 and *p* = 0.011, respectively). The Firmicutes/Bacteroidetes (F/B) ratio was considerably decreased in the CSP100 group (Figure 2E, *p* = 0.004). Furthermore, no substantial variations were observed in the relative abundances of Firmicutes and Bacteroidetes or the F/B ratio between the CON and CSP50 groups (*p* > 0.05). Figure 2B illustrates the top 15 microbial genera by relative abundance at the genus level in the colon. It was observed that *g_Prevotella*, *g_Clostridium,* and *g_Ruminococcus* were dominant across all colonic microbiota samples. Relative to the CON, *g_Prevotella* was significantly abundant in the CSP100 group (Figure 2F, *p* = 0.001), whereas the CSP50 group indicated a non-significant but decreasing trend. Moreover, relative to both the CON and CSP100 groups, *g_Ruminococcus* was substantially elevated in the CSP50 group (Figure 2H, *p* = 0.001). The abundance of *g_Clostridium* demonstrated no significant differences between the three groups (Figure 2G, *p* = 0.551).

Analysis of alpha diversity in microbial communities revealed that compared to the CON, the Shannon index and the Simpson index both increased in the CSP50 group, while the ACE index decreased. These changes did not reach statistical significance (Figure 2I, all *p* values > 0.05), indicating that CSP50 supplementation had no effect on microbial abundance and diversity. In contrast to the CSP50 group, the CSP100 group exhibited decreasing trends in the Chao1 index, the Simpson index, the Shannon index, and the ACE index, though all differences were non-significant (all *p* > 0.05). This indicates no changes in the abundance, species richness, or diversity of the gut microbiota in weaned piglets. Furthermore, PCoA based on the Bray–Curtis distance (Figure 2J) revealed distinct differences among the three groups along the PC1 (25.2%) and PC2 (14.5%) axes, indicating significant microbial differences between groups (*p* = 0.001). This suggests that providing weaned pigs with various protein sources generated unique microbial populations in the intestinal chyme.

### 3.4. KEGG Pathway Analysis of Colonic Microbial Genes

**Figure 3:**
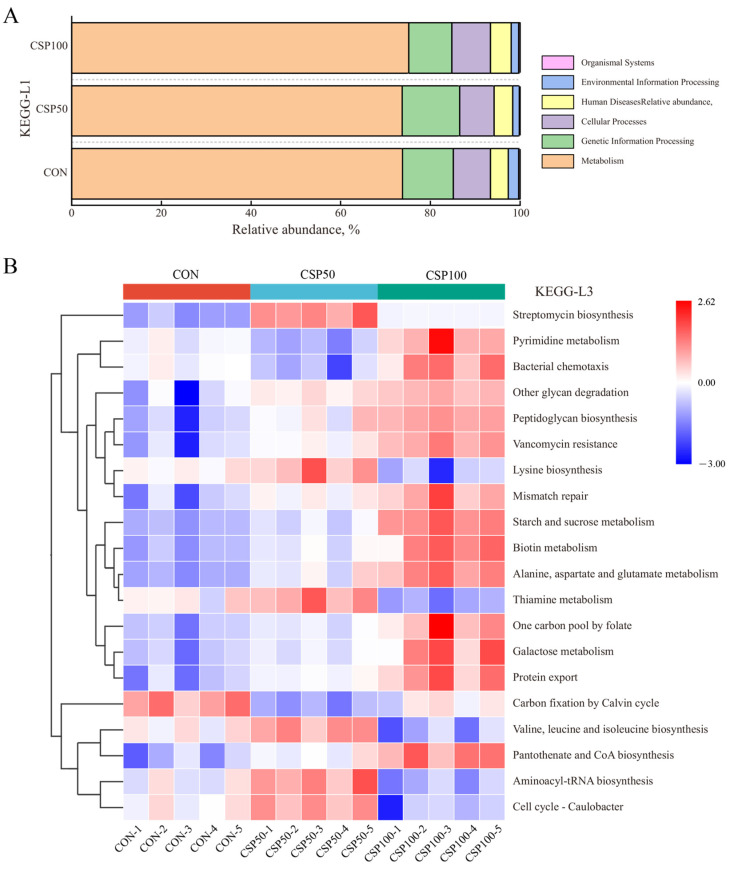
Functional Annotation of Microbial Communities in the Colonic Content. (**A**) Based on KEGG-L1 levels and (**B**) based on KEGG-L3 levels.

KEGG pathway analysis of each colonic microbial gene indicated that these genes were associated with 177 KEGG pathways distributed across six categories (Figure 3A). The gene abundances related to metabolic processes in the CON, CSP50, and CSP100 groups were 73.79%, 73.72%, and 75.20%, respectively. Among them, genes pertaining to Organismal systems demonstrated the lowest abundance, whilst those linked to Metabolic processes indicated a progressive increase.

At the KEGG-L3 level, statistical analysis of the top 20 functional genes in relative abundance within the colon microbiota indicated significant disparities in gene abundance across treatment groups (Figure 3B). The CON indicated the highest abundance of genes linked to carbon fixation by the Calvin cycle pathway. In the CSP50 group, there were significant increases in genes associated with Streptomycin biosynthesis, Lysine biosynthesis, Thiamine metabolism, Valine, leucine, and isoleucine biosynthesis, Aminoacyl-tRNA biosynthesis, and the Cell cycle-Caulobacter. Moreover, compared to the control group, the CSP50 and CSP100 groups, the abundance of genes in pathways associated with pyrimidine metabolism, bacterial chemotaxis, and pantothenate and CoA biosynthesis increased, peaking in the CSP100 group; however, genes related to carbon fixation by the Calvin cycle decreased.

### 3.5. Analysis Based on Species and Functional Differences and Microbial Association Network

**Figure 4:**
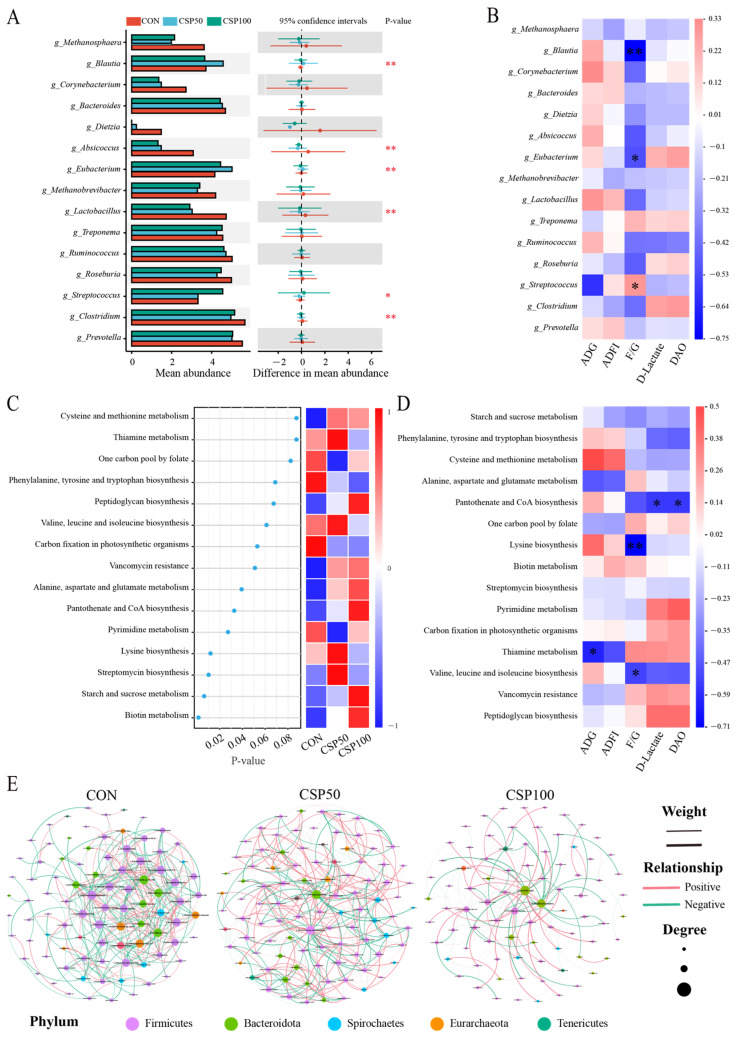
Effects of CSP on microbial species diversity and functional composition in the colonic chyme of weaned piglets. (**A**) Analysis of differences in genus-level abundance among the top 15 microbial species in colonic chyme (LDA ≥ 2). (**B**) Heatmap illustrating the correlation between distinct microorganisms and phenotypes. (**C**) Heatmap of differentially expressed KEGG metabolic pathways (*p* ≤ 0.1). (**D**) Correlation heatmap between differentially expressed KEGG metabolic pathways and phenotypes. Spearman’s correlation analysis was utilized. Red signifies a positive correlation, blue denotes a negative correlation, and the color intensity represents the strength of the association. (**E**) Visualization of microbial association networks. * *p* < 0.05, ** *p* < 0.01.

To identify species differences between the groups, the top 15 microbial genera in colonic chyme were analyzed by abundance (Figure 4A). The results indicated significant differences in *g_Blautia*, *g_Absicoccus*, *g_Eubacterium*, *g_Lactobacillus*, *g_Streptococcus,* and *g_Clostridium*. The CON indicated significantly higher abundance of *g_Blautia* and *g_Eubacterium*, and their abundance was significantly negatively correlated with F/G. Furthermore, the abundance of *g_Streptococcus* was substantially higher in the CSP100 group than in the CON and CSP50 groups, indicating a significant positive correlation with F/G (Figure 4B). This study visualized the top 15 microbial community functions ranked by relative abundance via the KEGG database L3 functional-level differential analysis (Figure 4C). The metabolic pathways of lysine biosynthesis and valine, leucine, and isoleucine biosynthesis exhibited negative correlations with F/G, thiamine metabolism was negatively associated with ADG, and pantothenate, while CoA biosynthesis was negatively related to DAO and D-lactate (Figure 4D). The microbial association network analysis identified the structural organization and interactions of the colonic chyme microbiota (Figure 4E, Table S1). Various proportions of CSP substituting soybean meal demonstrated unique complexities in microbial networks. In comparison to the CON, the CSP50 group demonstrated increases in total edges, total nodes, proportion of positive correlations, and clustering coefficient, whereas the CSP100 group showed the opposite trends. These findings suggest that the CSP50 group had improved overall complexity of the colonic microbiota network, while the CSP100 group had a marked reduction in network complexity.

## 4. Discussion

In pig farming, soybean meal serves as the primary protein source; however, its cost remains persistently high [18,19]. Therefore, identifying sustainable and cost-effective alternative protein sources to reduce dependence on soybean meal is crucial for maintaining the economic viability and resource sustainability of livestock production. CSP is considered a viable alternative due to its abundant yield and comparatively low expense. However, its anti-nutritional components (such as gossypol) can negatively impact weaned piglets at crucial phases of intestinal and immunological development, posing a limitation to its safe and effective use. This study systematically elucidated the dose–response effects of varying CSP replacement levels on weaned piglets and investigated their underlying mechanisms by integrating metagenomics technology with multidimensional indicators, including growth performance, carcass traits, organ indices, meat quality, intestinal barrier function, intestinal morphology, and gut microbiota structure and function.

The results indicated no significant differences in ADG, ADFI, and F/G between weaned piglets supplemented with various CSP doses, which is consistent with previous research [20]. It has been observed that substituting fish meal with 4% cottonseed protein at an equivalent ratio does not significantly impair the growth performance of weaned piglets but helps reduce nitrogen emissions in manure per kilogram of weight gain [14]. Moreover, replacing soybean meal with cottonseed meal at varying ratios (0%, 20%, 40%, 60%) in the diets of growing pigs, supplemented with enzyme preparations, demonstrated no adverse impacts on growth performance [21,22]. At appropriate supplementation doses, CSP can function as an efficient feed protein source, significantly decreasing soybean meal consumption and thereby reducing feed expenses. Here, the organ index analysis demonstrated that CSP did not significantly influence the organ indices of the hearts, livers, spleens, lungs, and kidneys of pigs, which is consistent with the growth performance. Previous studies have indicated that when dietary amino acids and energy meet nutritional requirements, the influence of protein levels on carcass traits and meat quality is relatively limited [23,24,25,26,27]. This study found no significant differences across groups in slaughter yield, carcass straightness, carcass diagonal length, average backfat thickness, or eye muscle area. However, the CSP group had a significantly decreased carcass weight. This reduction could result from intestinal injury that impairs protein deposition and nutritional absorption efficiency, thereby restricting intramuscular fat accumulation. This mechanism can substantially increase L*45 min and b*24 h. Moreover, Changes in L* and b* values are typically associated with myoglobin oxidation and lipid peroxidation in necropsy muscle, this study did not directly measure oxidative stress biomarkers, intramuscular fat, or gossypol residues. Therefore, the specific mechanism by which cottonseed protein intake induces intestinal injury and oxidative stress through color changes remains hypothetical and requires validation through measurement of oxidative stress biomarkers, intramuscular fat, or gossypol residues. These findings show the application potential of CSP in practical diets, as indicated by its beneficial effects on growth performance, carcass characteristics, and meat quality indicators.

The integrity of the intestinal barrier is essential for sustaining homeostasis in the body. Its role is to inhibit the infiltration of harmful agents, including bacteria, toxins, and antigens, from the intestinal lumen into the circulatory system via the mucosa [28]. Weaning stress can impair the intestinal mucosal barrier, usually resulting in compromised structural integrity and enhanced permeability [29], which leads to intestinal wall thinning and elevated intestinal permeability [30,31]. Serum DAO and D-lactate concentrations directly indicate the extent of intestinal permeability [32,33]. Previous research indicates that supplementing low-protein diets with 4% concentrated de-oiled hydrolyzed CSP and optimizing amino acid profiles will decrease serum DAO levels in weaned piglets [27]. Here, it was found that the CSP50 group had significantly decreased serum DAO activity and D-lactate levels, suggesting that this intervention enhances intestinal barrier function and reduces intestinal permeability. The integrity of intestinal morphology is essential for sustaining proper intestinal function [34]. Elevated VH often signifies an increased surface area for nutrient absorption, thus improving transport efficiency. However, certain studies have revealed that there is no substantial variation in VH or V/C ratio in the CSP cohort [35]. However, some studies have also indicated that there is no significant difference in villus height or villus-crypt ratio within the CSPID group [36]. This study demonstrates that, in comparison to the CON, the CSP100 group had decreased VH and V/C ratios in both the duodenum and ileum, but substantially increased CD in the jejunum. This indicates a reduction in the intestinal absorption surface and exacerbated mucosal injury, which inhibits digestive and absorptive functions. The CSP50 group showed no significant variations in intestinal morphology, VH, CD, or V/C ratios, indicating that moderate intake of CSP can preserve the integrity of intestinal structure. The activity of intestinal digestive enzymes directly indicates the ability to digest and absorb nutrients [37]. Alterations in this activity are frequently associated with the integrity of the intestinal mucosal structure and function. The pancreas secretes digestive enzymes, including amylase, lipase, and trypsin, which are essential for digestion by decomposing carbohydrates, lipids, and proteins [38]. Moreover, disaccharidases located on the brush border membrane of the small intestine facilitate the degradation and assimilation of carbohydrates [39,40]. Here, it was observed that the CON and CSP50 groups had significantly elevated duodenal mucosal lipase and jejunal lactase activity relative to the CSP100 group. Prior studies have shown that injury to the intestinal mucosa can impair digestive and absorptive functions, resulting in decreased activities of digestive enzymes, including maltase and amylase, which subsequently impacts nutrient absorption efficacy and intestinal health [36]. In this study, invertase activity in the duodenum and jejunum, as well as maltase activity in the ileum, was significantly elevated in the CSP50 group relative to both the CON and CSP100 groups. These data indicate that CSP50 consumption may preserve intestinal barrier and structural integrity, while CSP100 consumption may compromise intestinal health due to the accumulation of anti-nutritional components.

The gut microbiota is essential for animal health, with nutrition being a main factor influencing its composition and function [5,41]. This study performed metagenomic sequencing to examine the colonic microbiota composition in weaned pigs subjected to varying CSP doses. In pigs, the weaning transition represents a critical window during which dietary changes dramatically restructure the gut microbial ecosystem, as demonstrated by Chen et al. [42]. Among the predominant phyla in the mammalian gut, Bacteroidetes and Firmicutes play pivotal roles in energy harvest and host physiology. Bacteroidetes are recognized for their capacity to degrade complex polysaccharides and modulate immune responses through short-chain fatty acid production [43]. While Firmicutes encompass a diverse array of genera—including Blautia, Roseburia, and Faecalibacterium—that are key butyrate producers and contributors to metabolic regulation [44]. Moreover, it was observed that CSP did not alter the composition of these dominant bacterial phyla but significantly changed their relative abundances. The relative abundance of the Firmicutes phylum substantially decreased in the CSP100 group, whereas that of the Bacteroidetes was significantly increased, resulting in a significant decrease in the F/B ratio. This change is frequently associated with inflammatory bowel disease or alterations in dietary fiber fermentation patterns. The microbial community structure demonstrated dose-dependent variations at the species level. The relative abundance of *g_Ruminococcus* was significantly increased in the CSP50 group. Studies indicate that *g_Ruminococcus* synthesizes butyrate, which is advantageous to the host [45]. This bacterial genus is a crucial degrader of complex plant polysaccharides, and its abundance usually indicates a robust fiber fermentation process and improved microbial interaction networks. This alteration may align with the observed increase in intestinal permeability (reduced DAO and D-lactate levels), indicating its role in sustaining intestinal homeostasis. The CSP100 group demonstrated a substantial increase in *g_Prevotella* and did not indicate dominance of *g_Ruminococcus* [46,47]. This disrupting the normal microecological balance in the piglets’ intestines [48], causing impaired barrier function and immune dysfunction in the porcine intestinal mucosa [49]. This finding corresponds with the observations of intestinal morphological impairment (decreased VH and V/C ratio), elevated permeability (increased levels of DAO and D-lactate), and reduced digestive enzyme activity. The results indicate that the CSP50 group may have an additional benefit over the CSP100 group in preserving colon health, in the group supplemented with cottonseed meal, the proportions of Lactobacillus and Clostridium genera were slightly higher than in the groups supplemented with soybean meal and fish meal. 100% cottonseed meal significantly influenced the gut microbiota of weaned piglets, thereby affecting intestinal health outcomes [50]. Furthermore, the examination of microbial α-diversity indicated an increase in both bacterial richness and diversity within the CSP50 group. Moreover, PCoA revealed significant differences in microbial community structure between the CON and CSP groups.

Here, CSP was found to alter the gut microbial community structure. The differential analysis identified the following key species: *g_Blautia*, *g_Eubacterium*, and *g_Streptococcus*. As a dominant genus in the gut microbiota, *g_Blautia* plays a role in metabolic diseases, inflammatory conditions, and biotransformation [51]. *g_Blautia* and *g_Eubacterium*, also known as butyrate producers, belong to the Firmicutes phylum and are short-chain fatty acid bacteria. They serve as two major pillars of a healthy gut microbiome. The CSP50 group indicated significantly elevated abundance of *g_Blautia* and *g_Eubacterium*, which was significantly negatively correlated with the F/G ratio. This indicates that moderate CSP supplementation may augment the ability for intestinal butyrate synthesis by promoting butyrate-producing bacteria. Butyrate functions as the principal energy substrate for colonic epithelial cells and is crucial for epithelial cell proliferation, differentiation, intestinal barrier integrity, and mucosal immune regulation [52]. Moreover, it may improve energy acquisition efficiency in the colons of weaned piglets, thus optimizing overall feed conversion rates. However, *g_Streptococcus* abundance was highest in the CSP100 group, and it was substantially positively correlated with F/G, suggesting that high CSP intake may increase antinutritional factors (such as gossypol), thus promoting gut dysbiosis or a low-grade inflammatory state. This promotes the abundance of opportunistic infections and represents a risk of intestinal inflammation and impaired barrier function. Differences in microbial species can influence the functional capacity of microbial communities [53]. KEGG serves as an encyclopedia of genes and genomes, offering functional importance at the molecular level and beyond [54]. This study conducted functional annotation utilizing the KEGG database, uncovering active metabolic pathways. Moreover, STAMP analysis indicated negative associations between F/G ratios and the biosynthetic pathways of lysine, valine, leucine, and isoleucine. The increased activity of these key amino acid synthesis pathways indicates a gastrointestinal condition marked by elevated metabolism, robust development, and ecological equilibrium. This not only facilitates the rapid growth of advantageous bacteria but also indicates that their metabolites, including branched-chain amino acids, can help maintain gut health. Thiamine metabolism had an inverse relationship with ADG, indicating that microbes may enhance the host’s energy metabolism efficiency via endogenous vitamin supply, thus prompting physiological feeding regulation. However, the negative relationships between pantothenate and CoA biosynthesis with DAO and D-lactate levels may indicate that elevated CSP levels can alter the microenvironment, such that it compromises barrier integrity. In addition to investigating species and functional variations, this study further evaluated the effects of CSP supplementation on the ecological network of the gut microbiota. The gut microbiota constitutes a complex ecological network that plays a crucial role in maintaining host health [55,56]. The moderate CSP supplementation altered the microbial community structure and increased the complexity of microbial networks. This structural modification enhances the stability of the gut microbiota. Previous research has demonstrated that keystone species are essential for preserving the structural integrity of microbial ecological networks and the functional equilibrium of the host gut [57,58,59]. However, the mechanisms that govern the roles of these keystone species within the gastrointestinal network have yet to be comprehensively explained.

## 5. Conclusions

This study demonstrates that CSP can effectively replace soybean meal in weaned piglet diets without compromising growth performance. The effect of CSP exhibits a dose-dependent relationship. At moderate replacement levels (CSP50), supplementation altered gut microbiota composition (enriching *g-Lactobacillus* and *g-Brachybacterium*), increased microbial coexistence network complexity, improved intestinal morphology (increased jejunal VH and V/C), and reduced serum markers of intestinal permeability (DAO and D-lactic acid). Complete replacement (CSP100) produced opposite effects. These findings indicate that moderate CSP supplementation modulates the intestinal ecosystem and structure by enhancing barrier function, highlighting the importance of dose selection for practical applications. Therefore, moderate replacement doses are advised for practical implementation to attain the synergistic enhancement of production performance and intestinal health.

## Figures and Tables

**Table 1:** Composition and nutrient levels of the base diet (air-dried basis, %).

Ingredient	Content (%)		
	CON	CSP50	CSP100
Corn	62.00	62.85	63.70
Vegetable oil	1.00	1.00	1.00
Soybean meal	16.00	8.00	0
Full-fat soybean	10.00	9.00	8.00
Cottonseed protein	0	8.00	16.00
Fish meal	1.00	1.00	1.00
Wheat bran	3.00	3.00	3.00
Weaning King	4.00	4.00	4.00
3%Weaning King Premixture ^(1)^	3.00	3.00	3.00
L-lysine	0	1.50	3.00
Total	100	100	100
Nutrient levels ^(2)^			
Metabolic energy (Kcal/kg)	2918.42	2728.72	2552.52
Crude protein, %	17.14	17.26	17.16
Ca, %	1.47	1.46	1.44
Available P, %	0.92	1.00	1.09
Digestible Lys, %	0.65	0.52	0.63

^(1)^ The premixture provided the following per kg of diets: VA-12 400 IU, VB-1 3 mg, VB-2 10 mg, VB-5 15 mg, VB-6 8 mg, VB-12 0.04 mg, VD-3 2800 IU, VE 30 mg, VK-3 5 mg, Biotin 0.08 mg, Folic Acid 1 mg, D-Pantothenic Acid 15 mg, Nicotinic Acid 40 mg, Cu 16 mg, Fe 120 mg, Mn 70 mg, Zn 80 mg, I 0.70 mg, and Se 0.48 mg. ^(2)^ Metabolic energy and amino acid contents were assessed using the NRC requirement values, and the levels of other nutrients were also measured. CON = control; CSP50 = 50% cottonseed protein and 50% soybean meal; CSP100 = 100% cottonseed protein.

**Table 2:** Effects of replacing soybean meal with CSP on the growth performance, carcass traits, organ indices and pork quality in weaned piglets.

Items	Groups			SEM	*p*-Value		
	CON	CSP50	CSP100		ANOVA	Linear	Quadratic
IBW, kg	9.61	9.66	9.64	0.22	0.432	0.279	0.279
FBW, kg	20.77	20.92	20.30	0.15	0.218	0.193	0.231
Day 1–14							
ADG, g	349.63	355.18	329.13	6.37	0.226	0.194	0.243
ADFI, g	508.44	523.16	501.27	10.01	0.703	0.439	0.439
F/G	1.46	1.47	1.52	0.01	0.259	0.121	0.646
Day 15–28							
ADG, g	448.07	448.63	432.70	5.73	0.478	0.310	0.521
ADFI, g	708.75	713.38	696.37	7.34	0.548	0.523	0.382
F/G	1.58	1.60	1.61	0.01	0.633	0.358	0.882
Day 1–28							
ADG, g	398.85	401.91	380.91	5.25	0.224	0.171	0.279
ADFI, g	608.60	620.27	598.82	6.83	0.482	0.291	0.291
F/G	1.53	1.55	1.57	0.01	0.176	0.071	0.856

Data are shown as the mean with the SEM (*n* = 5). IBW = initial body weight; FBW = final body weight; ADG = average daily gain; ADFI = average daily feed intake; F/G = feed conversion ratio. CON = control; CSP50 = 50% cottonseed protein and 50% soybean meal; CSP100 = 100% cottonseed protein.

**Table 3:** Effects of replacing soybean meal with CSP on the carcass traits in weaned piglets.

Items	Groups			SEM	*p*-Value		
	CON	CSP50	CSP100		ANOVA	Linear	Quadratic
Carcass weight, kg	14.55 ^a^	13.93 ^b^	13.60 ^b^	0.15	0.014	0.005	0.551
Carcass yield, %	51.16	50.25	50.28	0.42	0.634	0.424	0.617
Carcass straight length, cm	67.00	65.20	63.80	1.21	0.590	0.315	0.941
Carcass oblique length, cm	63.00	62.00	60.00	1.18	0.608	0.337	0.851
Average backfat thickness, mm	9.75	9.40	9.53	0.17	0.722	0.611	0.540
Eye muscle area, cm^2^	19.51	18.52	18.97	0.30	0.442	0.291	0.291

Data are shown as the mean with the SEM (*n* = 5). ^a, b^ Different superscripts represent a significant difference (*p* < 0.05). CON = control; CSP50 = 50% cottonseed protein and 50% soybean meal; CSP100 = 100% cottonseed protein.

**Table 4:** Effects of replacing soybean meal with CSP on the organ indices in weaned piglets.

Items	Groups			SEM	*p*-Value		
	CON	CSP50	CSP100		ANOVA	Linear	Quadratic
Heart, %	4.37	4.56	4.69	0.10	0.456	0.221	0.876
Liver, %	18.97	17.89	19.72	0.47	0.288	0.155	0.155
Spleen, %	1.72	1.79	1.77	0.03	0.588	0.473	0.468
Lung, %	11.05	10.89	11.36	0.17	0.540	0.475	0.402
Kidney, %	3.52	3.59	3.64	0.03	0.301	0.132	0.831

Data are shown as the mean with the SEM (*n* = 5). CON = control; CSP50 = 50% cottonseed protein and 50% soybean meal; CSP100 = 100% cottonseed protein.

**Table 5:** Effects of replacing soybean meal with CSP on the pork quality in weaned piglets.

Items	Groups			SEM	*p*-Value		
	CON	CSP50	CSP100		ANOVA	Linear	Quadratic
Longissimus							
pH 45 min	6.23	6.25	6.16	0.02	0.202	0.198	0.204
pH 24 h	5.71	5.72	5.62	0.02	0.172	0.139	0.233
L* 45 min	36.49 ^b^	39.68 ^a^	39.73 ^a^	0.46	0.002	0.002	0.073
a* 45 min	15.24	14.21	14.45	0.34	0.456	0.359	0.394
b* 45 min	9.75	9.85	10.37	0.16	0.214	0.104	0.517
L* 24 h	39.61	42.15	41.50	0.62	0.232	0.221	0.231
a* 24 h	21.28	19.45	21.78	0.62	0.275	0.118	0.118
b* 24 h	30.06 ^b^	35.26 ^a^	36.22 ^a^	0.86	0.005	0.003	0.210
Cooking water loss, %	34.03	35.46	36.76	0.94	0.535	0.273	0.975
Shear force, N	26.52	23.69	24.18	0.97	0.482	0.354	0.444
Drip loss, %	2.96	3.52	3.23	0.25	0.698	0.691	0.462

Data are shown as the mean with the SEM (*n* = 5). ^a, b^ Different superscripts represent a significant difference (*p* < 0.05). CON = control; CSP50 = 50% cottonseed protein and 50% soybean meal; CSP100 = 100% cottonseed protein.

**Table 6:** Effects of replacing soybean meal with CSP on intestinal permeability in weaned piglets.

Items	Groups			SEM	*p*-Value		
	CON	CSP50	CSP100		ANOVA	Linear	Quadratic
D-Lactate, μmol/L	64.18 ^b^	49.27 ^c^	80.96 ^a^	3.09	<0.001	0.002	0.001
DAO, ng/mL	6.55 ^b^	4.81 ^c^	7.86 ^a^	0.29	<0.001	0.007	0.005

Data are shown as the mean with the SEM (*n* = 5). ^a, b, c^ Different superscripts represent a significant difference (*p* < 0.05). CON = control; CSP50 = 50% cottonseed protein and 50% soybean meal; CSP100 = 100% cottonseed protein.

**Table 7:** Effects of replacing soybean meal with CSP on intestinal morphology in weaned piglets.

Items	CON	CSP50	CSP100	SEM	*p*-Value		
					ANOVA	Linear	Quadratic
Duodenum							
VH, μm	505.95 ^a^	504.21 ^a^	470.34 ^b^	7.25	0.047	0.028	0.182
CD, μm	357.09	362.76	388.67	7.12	0.152	0.075	0.457
V/C	1.42 ^a^	1.39 ^a^	1.21 ^b^	0.04	0.028	0.014	0.204
Jejunum							
VH, μm	400.00	396.36	364.27	11.80	0.454	0.268	0.595
CD, μm	275.16 ^b^	273.92 ^b^	296.95 ^a^	4.54	0.033	0.024	0.102
V/C	1.45	1.45	1.23	0.05	0.169	0.101	0.333
Ileum							
VH, μm	399.28 ^a^	393.30 ^a^	364.38 ^b^	5.94	0.006	0.003	0.111
CD, μm	244.38	251.58	259.38	4.73	0.494	0.254	0.254
V/C	1.64 ^a^	1.57 ^a^	1.41 ^b^	0.04	0.019	0.007	0.403

Data are shown as the mean with the SEM (*n* = 5). ^a, b^ Different superscripts represent a significant difference (*p* < 0.05). CON = control; CSP50 = 50% cottonseed protein and 50% soybean meal; CSP100 = 100% cottonseed protein.

**Table 8:** Effects of replacing soybean meal with cottonseed protein on mucosal enzyme activity in weaned piglets.

Items	CON	CSP50	CSP100	SEM	*p*-Value		
					ANOVA	Linear	Quadratic
**Duodenum**							
Invertase, U/mL	42.60 ^b^	45.15 ^a^	40.14 ^b^	0.78	0.018	0.122	0.012
Maltase, U/L	107.16	123.54	105.11	4.32	0.164	0.839	0.064
Lactase, U/mL	10.67	10.92	9.27	0.34	0.086	0.077	0.155
Trypsin, U/L	226.07	206.18	183.53	7.61	0.061	0.021	0.021
Lipase, nmol/min/g	444.80 ^a^	431.04 ^a^	326.94 ^b^	16.88	<0.001	<0.001	0.056
α-Amylase, 10 mg/30 min/g	15.66	14.42	13.09	0.57	0.194	0.076	0.973
**Jejunum**							
Invertase, U/mL	83.40 ^b^	94.77 ^a^	78.55 ^b^	2.08	<0.001	0.093	<0.001
Maltase, U/L	93.99	108.82	87.08	4.40	0.683	0.812	0.812
Lactase, U/mL	15.32 ^a^	15.34 ^a^	11.77 ^b^	0.50	<0.001	<0.001	0.004
Trypsin, U/L	177.76	170.43	167.63	3.99	0.600	0.337	0.800
Lipase, nmol/min/g	561.53	548.85	512.66	13.80	0.349	0.170	0.692
α-Amylase, 10 mg/30 min/g	16.76	16.46	15.36	0.38	0.298	0.146	0.616
**Ileum**							
Invertase, U/mL	59.18 ^ab^	66.13 ^a^	56.99 ^b^	1.45	0.013	0.429	0.005
Maltase, U/L	79.83 ^b^	103.17 ^a^	77.79 ^b^	4.38	0.017	0.808	0.005
Lactase, U/mL	15.33	14.36	12.95	0.45	0.085	0.030	0.030
Trypsin, U/L	142.37	142.97	119.95	4.65	0.057	0.039	0.184
Lipase, nmol/min/g	345.71	336.50	289.53	10.53	0.051	0.024	0.336
α-Amylase, 10 mg/30 min/g	16.81	16.41	14.39	0.50	0.097	0.046	0.405

Data are shown as the mean with the SEM (*n* = 5). ^a, b^ Different superscripts represent a significant difference (*p* < 0.05). CON = control; CSP50 = 50% cottonseed protein and 50% soybean meal; CSP100 = 100% cottonseed protein.

## Data Availability

Data is contained within the article or Supplementary Materials.

## Supplementary Materials

The following supporting information can be downloaded at https://www.mdpi.com/article/10.3390/ani16060946/s1. Table S1: Topological Features of the Colon Microbiome Network.
