# Supplementary material for: Dose-Dependent Effects of Replacing Soybean Meal with Cottonseed Protein: Key to Optimizing Gut Health in Weaned Piglets
Source: Animals (Basel). 2026 Mar 18;16(6):946. doi: 10.3390/ani16060946 (PMC13023351; doi:10.3390/ani16060946)
Supplement: Supplementary file 1 [file animals-16-00946-s001.zip › animals-4158167-supplementary.pdf]

Table S1. Topological Features of the Colon Microbiome Network.

Index	CON	CP50	CP100
Total nodes	90	92	84
Total links	309	382	128
Positive correlation ratio	54.69%	57.59%	55.47%
Negative correlation ratio	45.31%	42.41%	44.53%
Average degree	6.876	7.796	3.048
Average path length	3.227	2.714	3.642
Clustering coefficient	0.316	0.414	0.239
